# Simulation study of a novel small animal FLASH irradiator (SAFI) with integrated inverse-geometry CT based on circularly distributed kV X-ray sources

**DOI:** 10.1038/s41598-023-47421-0

**Published:** 2023-11-17

**Authors:** Yuewen Tan, Shuang Zhou, Jonathan Haefner, Qinghao Chen, Thomas R. Mazur, Arash Darafsheh, Tiezhi Zhang

**Affiliations:** 1 Department of Radiation Oncology, Washington University School of Medicine in St. Louis, St. Louis, MO 63110 USA; 2 Department of Physics, Washington University in St. Louis, St. Louis, MO 63130 USA; 3 Present Address: Center for Radiological Research, Columbia University, New York, NY 10032 USA

**Keywords:** Biomedical engineering, Radiotherapy, Design, synthesis and processing

## Abstract

Ultra-high dose rate (UHDR) radiotherapy (RT) or FLASH-RT can potentially reduce normal tissue toxicity. A small animal irradiator that can deliver FLASH-RT treatments similar to clinical RT treatments is needed for pre-clinical studies of FLASH-RT. We designed and simulated a novel small animal FLASH irradiator (SAFI) based on distributed x-ray source technology. The SAFI system comprises a distributed x-ray source with 51 focal spots equally distributed on a 20 cm diameter ring, which are used for both FLASH-RT and onboard micro-CT imaging. Monte Carlo simulation was performed to estimate the dosimetric characteristics of the SAFI treatment beams. The maximum dose rate, which is limited by the power density of the tungsten target, was estimated based on finite-element analysis (FEA). The maximum DC electron beam current density is 2.6 mA/mm^2^, limited by the tungsten target's linear focal spot power density. At 160 kVp, 51 focal spots, each with a dimension of $2\times 20$ mm^2^ and 10° anode angle, can produce up to 120 Gy/s maximum DC irradiation at the center of a cylindrical water phantom. We further demonstrate forward and inverse FLASH-RT planning, as well as inverse-geometry micro-CT with circular source array imaging via numerical simulations.

## Introduction

FLASH radiotherapy (FLASH-RT) at ultra-high dose rates (UHDRs) (> 40 Gy/s) has gained momentum since the publication of an in vivo study in 2014 by Favaudon et al*.*, demonstrating a significant reduction in normal tissue toxicity using FLASH-RT while maintaining similar tumor control when compared with conventional dose rate RT (~ 0.03 Gy/s)^1^. Multiple follow-up studies confirmed the FLASH effect and the first human patient was treated in 2019 using electron FLASH-RT^2–8^. Multiple clinical trials are ongoing worldwide.

There is intense research effort into understanding the biological mechanism of the FLASH effect, which remains hotly debated^2,9–14^. Pre-clinical research is of great importance in guiding FLASH-RT to clinical applications^15^. In order to fully explore the dependence of the FLASH effect on temporal characteristics of the radiation beam, FLASH irradiator platforms capable of delivering both conventional and UHDR with precise control over the delivered time are required. To date, nearly all FLASH pre-clinical studies have been performed using electron and proton beams, with the former constituting the vast majority. Electron FLASH beams have been produced using dedicated or modified linear accelerators (linacs)^16–18^. Proton FLASH beams have been produced using singletron, isochronous cyclotrons, and synchrocyclotrons^19–24^. Such modalities require high capital investment and maintenance, are mostly dedicated to specific experimental setups or patients' treatment, and are not accessible to most laboratory researchers.

Producing x-ray photon FLASH beams is more challenging due to the low efficiency of the Bremsstrahlung process. Although a few x-ray FLASH effect studies have been performed with kV and MV x-ray beams generated in national research centers, a compact and affordable FLASH-RT system is more compelling for pre-clinical photon FLASH-RT research^2,25^. Bazalova-Carter and Esplen studied the feasibility of achieving UHDR by a commercially available x-ray tube with a fixed anode^26^. Although the simulation suggests the dose rate can be as high as 160 Gy/s outside the tube housing, the dose rate falls off very fast due to its low x-ray mean energy. In a later development of a kV FLASH tube for in vitro irradiations, x-ray beams were controlled by a shutter to deliver FLASH irradiations in pulses^27^. Rezaee et al. proposed another kV FLASH design consisting of two commercial diagnostic x-ray tubes with rotating anodes facing each other^28^. The FLASH dose region in their design has a uniform dose rate distribution. Due to the limited space and size of the x-ray tube, it is impractical to employ more than two x-ray tubes in such a design.

In clinical intensity-modulated radiation therapy (IMRT), the radiation dose is delivered from multiple angles by the rotating gantry head of a linac. It has been proposed that PHASER, a multi-head linac system, can be designed for conformal MV photon FLASH-RT^29^. However, adopting a similar concept using kV sources for conformal FLASH-RT of small animals is not trivial, because of the bulky physical size of the x-ray sources and the close proximity to the source required to achieve a UHDR beam. It has not yet been possible to concurrently deliver FLASH-RT at multiple beam angles using conventional x-ray sources with fixed or rotating anodes.

The emerging distributed x-ray sources initially designed for x-ray imaging in image-guided radiotherapy provide an ideal solution for pre-clinical conformal x-ray FLASH-RT. These kilovoltage (kV) sources using powerful thermionic-emission cathode array generate a plurality of focal spots^30,31^. With all the focal spots within one vacuum envelope, the distributed x-ray source is compact enough to deliver FLASH-RT to an object close to the focal spots. Moreover, adding a detector array to the distributed x-ray source forms an inverse-geometry computed tomography (CT) through which high-resolution micro-CT images can be obtained.

This work presents a simulation study of the novel small animal FLASH irradiator (SAFI) based on the circular distributed kV x-ray source array with 51 focal spots.

## Materials and methods

### Overall SAFI system design

**Figure 1:**
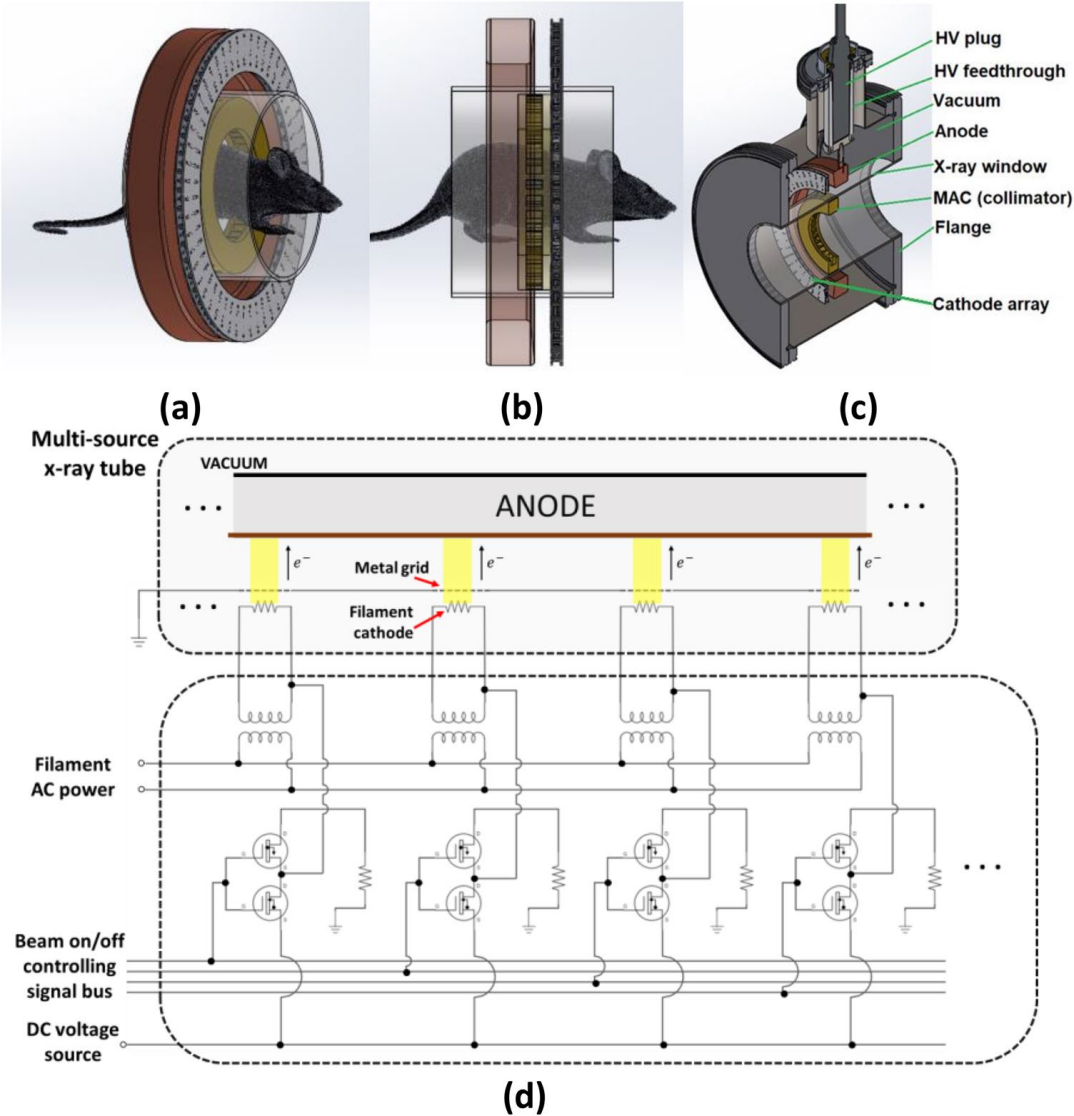
(**a**–**c**) Schematics of the SAFI system based on a circular x-ray source array. Fifty-one cathodes are equally distributed on a circle and generate a circular array of focal spots on a ring-shaped anode. A multi-aperture collimator (MAC), designed based on the specific anatomy of the animal subject in treatment planning, is positioned outside the vacuum envelope and collimates the beams to the target. (**d**) Schematic of the beam controller design based on the multi-source x-ray tube design (electron optics and electrode voltage details are omitted). All beams are turned on simultaneously and with optimized intensity during FLASH-RT treatment, while for inverse-geometry micro-CT imaging, they are turned on sequentially.

Figure 1a–c illustrates the design of the SAFI system, which comprises a fixed annular anode with a tungsten target and a circular array of cathodes in a vacuum housing. The small animal subject and a multi-aperture collimator (MAC) are located in the bore outside the vacuum housing. When activated, the constituent cathodes generate a circular array of focal spots on the targets. The current impinging to the anode on each focal spot is defined as the anode current. The bore inner diameter is 14 cm. The MAC collimates the x-ray beams to the treatment target. The design of the MAC is based on the geometry of the animal subject, with aperture shape and beam intensity varied in order to achieve dose distributions similar to clinical IMRT.

The same circular source array is also used for onboard micro-CT imaging when operating at lower kVp, smaller focal spots, and lower anode current. In imaging, the treatment MAC will be replaced with another MAC dedicated to imaging. Due to space limitations, a small area detector will be used forming an inverse-geometry CT^32^. The detector and MAC will rotate together about the animal subject while the sources that produce x-rays intercepting the imaging field-of-view are turned on sequentially.

A grounded mesh grid will be used to control the beam on and off in a manner similar to that used by the linear x-ray source that we developed previously^30^. When a cathode is connected to a negative bias voltage, thermionic electrons will pass through a grid mesh and generate an x-ray beam at the corresponding focal spot location on the tungsten target, as shown in Fig. 1d. Due to the complexity of the electron beam control, the SAFI system employs a unipolar design with the anode connecting to a positive high voltage and the mesh grid grounded. Each source can be addressed independently by a controller, and they can be turned on/off simultaneously for FLASH-RT, or sequentially for inverse-geometry micro-CT imaging.

### FLASH-RT treatment

**Figure 2:**
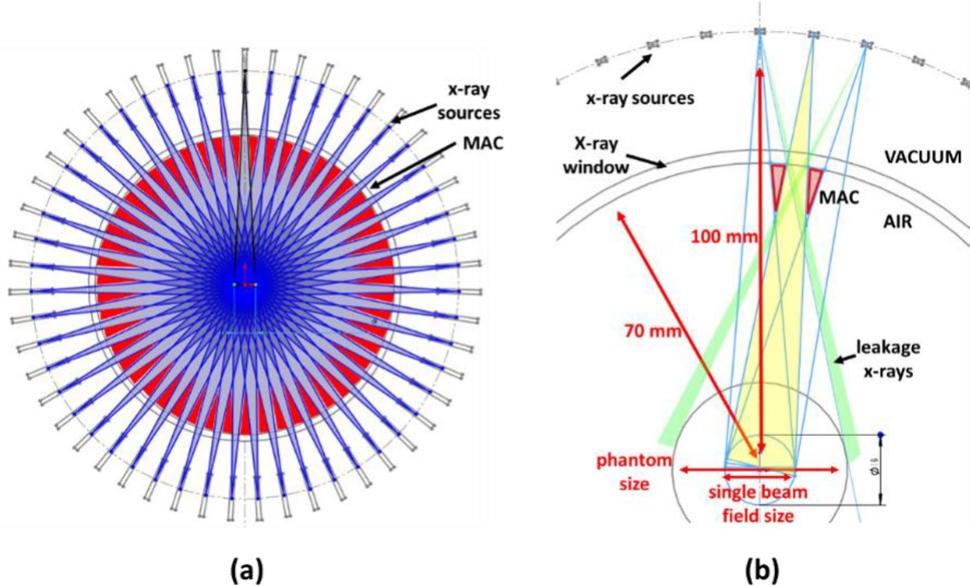
(**a**) Demonstration of SAFI geometry with 51 beams pointing to a circular target at the center. The red triangles represent MAC’s collimation areas. The shapes of the MAC apertures can be designed based on the anatomy of the animal subject. The MAC can be manufactured by 3D printing techniques. (**b**) Diagram of single-beam MAC design and illustration of x-ray leakage from the neighboring sources. A properly designed MAC should also prevent unnecessary radiation exposure by leaking x-rays from adjacent sources.

Figure 2a and b illustrate the geometry of SAFI system with 51 sources for a circular target and the corresponding design of MAC. Each individual MAC will be designed based on the shape of the particular target by treatment planning software and will be manufactured using 3D printing techniques. The MAC collimates x-ray beams to the target while preventing unnecessary radiation from leakage x-rays produced by adjacent sources. The maximum target size is jointly determined by the system geometry and the number of sources. As shown in Fig. 2b, x-ray beams may leak through the adjacent slots if treating an oversized target, resulting in undesired radiation exposure to the animals. The intensity and shape of each beam can be optimized by inverse planning to achieve the desired doses to target and normal tissue. The x-ray intensity of each beam can be controlled by the cathode beam current or a physical compensator.

### Inverse-geometry CT imaging

**Figure 3:**
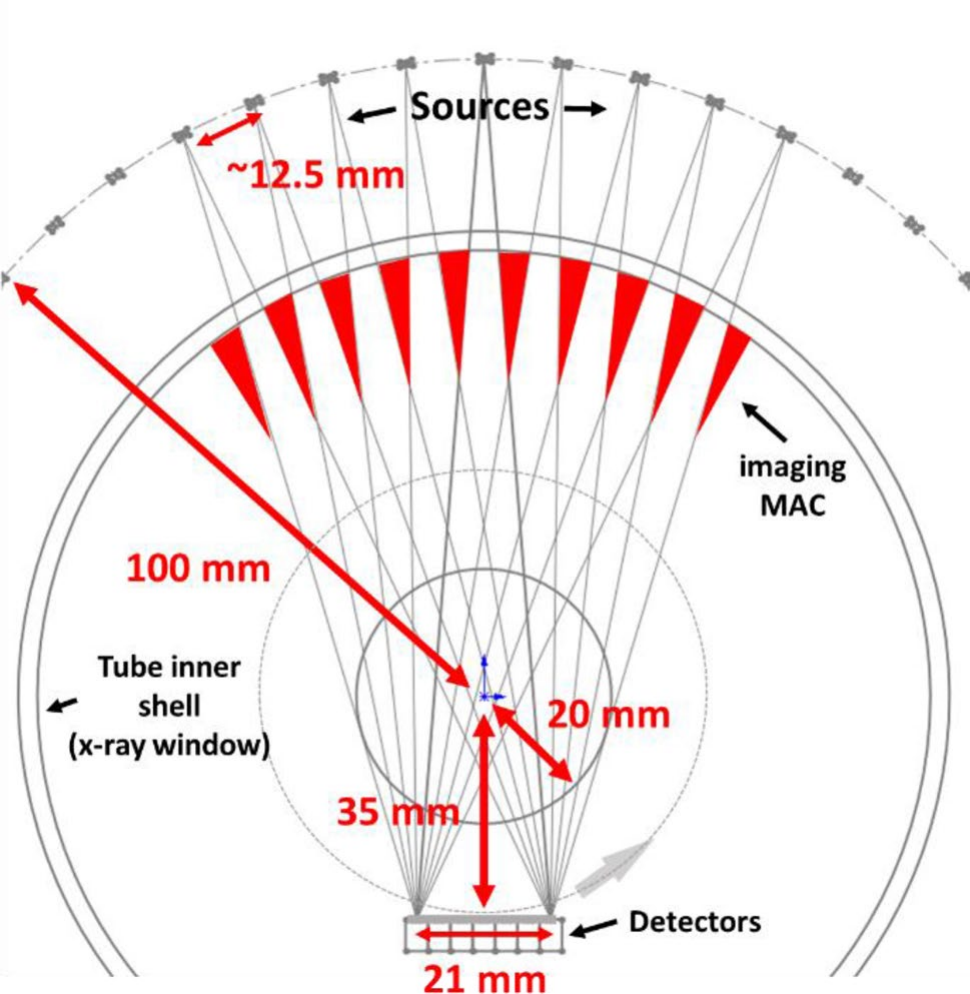
Diagram of inverse-geometry CT with rotatable detectors and dedicated imaging MAC. The red triangles represent the imaging MAC.

The small animal irradiator needs onboard imaging for treatment planning and setup. The same source array can also be used for onboard inverse-geometry CT imaging. The full-ring MAC for RT will be placed by a rotatable imaging structure, which comprises a flat small-area detector array and an imaging MAC as shown in Fig. 3. The imaging MAC is designed based on the detector shape in the beam-eye view direction. Similar to the treatment MAC, the imaging MAC should conform the x-ray beam to the detector. It can be made using a 3D printing method similar to the treatment MAC. The detector array and imaging MAC will rotate in steps during acquisition while the source array stays stationary such that the MAC can identically collimate beams for each set of projection data. At each step angle, the multi-source beam controller sequentially turns on or off the nine sources opposite to the detector array to fully sample the entire field of view. Without rotating the animal or the sources, a rotation of the detector alone will generate only 51 CT projection angles for image reconstruction. To reconstruct high-resolution images, we can repeatedly rotate the source array or the animal subject by a small step angle (less than the angular spacing between sources) to get a new set of CT sampling in another full detector rotation for acquiring more projection angles.

### Estimation of dose rate per incident electron beam current using Monte Carlo simulation

**Figure 4:**
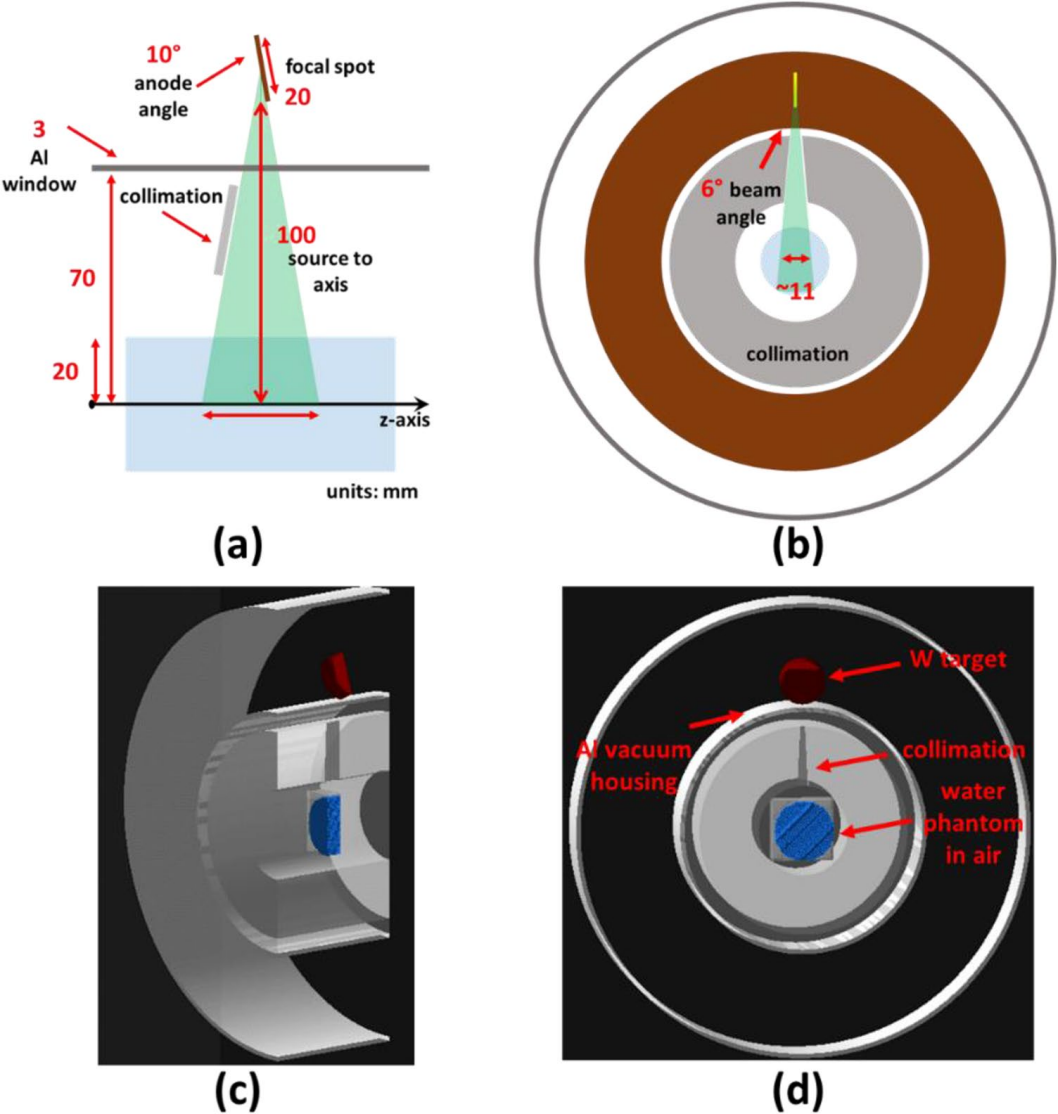
Sagittal (**a**) and transverse (**b**) views of the SAFI system geometry; (**c**) and (**d**) Geant4 model of a single source x-ray field for dose rate estimation based on (**a**) and (**b**) design.

Monte Carlo (MC) simulations were performed to evaluate the relation between the anode current and the dose rate in the phantom using Geant4 toolkit with the most precise low-energy electromagnetic physics package “G4EmStandardPhysics_option4”, which has been previously validated^33,34^. Figure 4a and b show the fan-beam geometry and collimation of a single x-ray source. (c) and (d) show the corresponding geometries in the Geant4 visualization. We modeled electron beams with 120, 160, and 200 keV energies that were incident on the tungsten target with a ${10}^{\circ }$ anode angle. The cylindrical water phantom with a diameter of 10, 20, or 40 mm was placed at the geometric center of the irradiator and voxelized by 1 mm^3^ cubes. The distance from the focal spots to the phantom center was 100 mm. Because of the low conversion rate of x-rays from electron bombardment, the simulation had been done in two steps: (1) the x-ray production before entering the water phantom, and (2) the dose distribution in a cylindrical water phantom.

In the first step, 2 million electrons were incident on the tungsten target in each run, and the produced x-rays were attenuated by the 3-mm Al tube housing. Before x-rays entered the water phantom, a virtual detector collected the x-ray photon fluence rates for each energy $E$ per unit anode current, based on the number of input electrons and denoted as $\dot{\Phi }\left(E\right)/I$. In the second step, 200 million uniformly distributed fan-beam x-ray photons of energy $E$ was used as input to the water phantom. The dose distributions per photon fluence $D(E)$ in the water phantom was obtained for each photon energy $E$. All simulation runs had sufficient number of input particles to get less than 5% of dose uncertainties in each voxel. The dose rate in the water phantom per unit anode current of a single electron source (${\dot{D}}_{i}/I$) can then be calculated based on the fluence rate energy spectrum $\dot{\Phi }\left(E\right)/I$ obtained from the first step, according to,

1 $$\frac{{\dot{D}}_{i}}{I}=\underset{0}{\overset{{E}_{max}}{\int }}\frac{\dot{\Phi }\left(E\right)}{I}\cdot D\left(E\right)\cdot dE,$$

in units of Gy/s/mA. To achieve a dose rate ${\dot{D}}_{FLASH}$ at the center of the phantom for *N* electron sources in the design, the anode current required for each source is simply

2 $${I}_{FLASH}=\frac{{\dot{D}}_{FLASH}}{N\cdot \frac{{\dot{D}}_{i}}{I}}$$

in units of mA.

### Maximum focal spot power density and estimation of focal spot size

As with other x-ray sources, the maximum power of the SAFI system is limited by the maximum focal spot power density of the electron beam, defined as the maximum electron beam heat power divided by the focal spot size. In order to estimate SAFI’s maximum power, finite-element analysis software (COMSOL, Inc., Burlington, MA) was used to calculate the transient temperature distribution in the target under electron bombardment. In the numerical simulation, the tungsten target of 1 cm thickness is attached on a large copper heat sink with infinite boundary on the back. The heat flux was used as the heat source on a line focal-spot area *A*. The area was set to be greater than 1 mm by 10 mm. In this case, the maximum temperature changes less than 0.1% for a fixed input power density. The physics-based tetrahedral meshes were created in the software, and the tungsten mesh size was less than 100 μm in depth and 2 mm in lateral directions. We estimated the maximum heat input power density ${P}_{in}/A$ that can be allowed with the line focal spot area *A*. The maximum incident current density for a focal spot was obtained by

3 $$\frac{{I}_{\text{max}}}{A}=\frac{{P}_{in}/A}{U}\cdot \frac{1}{{c}_{b}(U)}$$

in units of mA/mm^2^, where $U$ is the voltage difference between anode and cathode (160 kV in this study), and ${c}_{b}(U)$ is the backscattering coefficient of the corresponding energetic electron on solid tungsten. Following Eq. (2), the total area of the focal spot required to achieve FLASH dose rate ${\dot{D}}_{FLASH}$ is

4 $${A}_{Flash}=\frac{{I}_{FLASH}}{{I}_{max}/A}= \frac{{\dot{D}}_{FLASH}}{N\cdot \frac{{\dot{D}}_{i}}{I}}\cdot \frac{A\cdot U\cdot {c}_{b}\left(U\right)}{{P}_{in}} .$$

### FLASH-RT Forward and Inverse Treatment Planning

In the forward treatment planning, the central plane dose distribution was obtained from the MC calculation, smoothed, and linearly interpolated on a 1 µm^2^ grid. This result was used as kernels to get dose distributions for 51 sources by image rotation using the nearest-neighbor interpolation method.

Conventional IMRT plans are inversely optimized and delivered with multi-leaf collimator (MLC). The SAFI system can deliver IMRT by modulating the weight, fluence intensity, or aperture shape of each beam. We developed a simple beam-weight optimization method with an aperture shape determined by the target geometry. A cylindrical water phantom with a target in the center and two adjacent organs-at-risk (OARs) was used in treatment planning. The beam weights were optimized by the inverse optimization algorithm that minimizes the sum of the square of the difference between prescribed and actual doses. The inverse optimization is formulated as minimization of a two norm,

5 $${I}^{*}=\underset{I\ge 0}{\mathrm{argmin}} ||b-{A}_{d}I|{|}_{2},$$

where $I$ is the relative beam intensities of 51 sources, $b$ is the planning goal, and ${A}_{d}$ is the dose distribution mapping that evaluates the dose distribution from each beam. The solution was obtained by the pseudo-inverse method.

### Electron optics design

FLASH-RT requires large focal spots to achieve UHDR, while inverse-geometry CT needs smaller focal spots for high-resolution imaging. We designed the electron optics of the SAFI system that consisted of multiple focusing electrodes. The focal spot size can be changed by adjusting the voltage of the focusing electrodes. The finite-element charged-particle-tracing model in COMSOL was used for the electron optics design.

### Iterative image reconstruction

The source array of the SAFI system can remain stationary, and only the detector rotates. However, a stationary SAFI system can only produce 51 projection measurements. By rotating either the animal subject or the sources with *N* small-angle steps between the adjacent source angles, one can obtain $N$-fold projection angles that can be used to reconstruct high-resolution images. We implemented an iterative image reconstruction algorithm to reconstruct tomographic images from limited views^35^. With the system matrix ${A}_{s}$, the image data $\mu$ is reconstructed from the measurement $y$ by minimizing the objective function

6 $${\mu }^{*}= \underset{\mu \ge 0}{\mathrm{argmin}}({\left(y-{A}_{s}\mu \right)}^{T} D \left(y-{A}_{s}\mu \right)+\lambda {R}_{TV}\left(\mu \right))$$

where $D$ is a diagonal matrix associated with the Poissonian noise of the projection data, ${R}_{TV}\left(\mu \right)$ is the regularization term based on the prior knowledge of the total variation in adjacent locations, which has the form of ${R}_{TV}\left(\mu \right)= {\sum }_{i,j}\sqrt{{\left({\mu }_{i,j+1}-{\mu }_{i,j}\right)}^{2}+{\left({\mu }_{i+1,j}-{\mu }_{i,j}\right)}^{2}}$^36^, and λ is the tuning parameter to set the strength of the total variation regularization. The optimization was performed using the conjugate descent algorithm. We generated projection data using the FORBILD head phantom and compared the difference in image quality with different numbers of projection angles.

## Results

### Dosimetric characteristics of FLASH-RT beam

**Figure 5:**
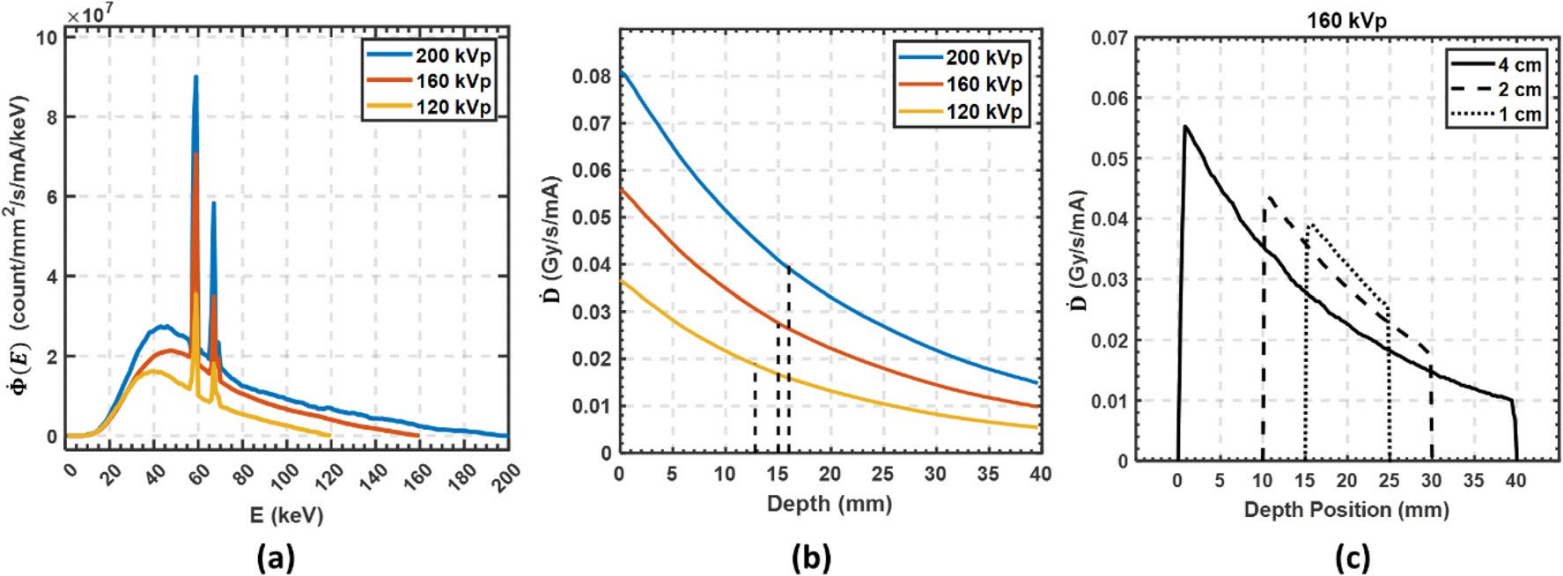
Monte Carlo simulation results: (**a**) Energy spectra of 120, 160, and 200 kVp beams per mA beam current. (**b**) The depth dose rate of 120, 160, and 200 kVp x-ray beams per mA of beam current in a 4-cm cylindrical water phantom. The half-value dose rates for three kVps are shown by dotted lines. (**c**) The 160 kVp depth dose rate in water phantom with diameters of 1 cm, 2 cm, and 4 cm. A smaller phantom receives higher dose rate at the center.

Figure 5a plots the fluence rate energy spectra $\dot{\Phi }\left(E\right)$ of three kVp values (120, 160, and 200) obtained by the Geant4 MC. As estimated by Eq. (1), the centerline depth dose rates per mA current for the same three kVps are plotted in Fig. 5b. The 50% of maximum dose rate for 120, 160, and 200 kVp are at depths of 12.6, 15.0 and 16.0 mm, respectively. The increase in the 50% depth dose from 160 to 200 kVp was marginal at approximately 1 mm. In order to ease the dielectric insulation requirements, the SAFI system will operate at 160 kVp for FLASH-RT treatment. Although the dose rate at the center per anode current of 200 kVp is about 30% higher than the 160 kVp, the higher kinetic energy also results in more energy deposition on the target, which limits its peak operating current. At 160 kVp, the dose rate per mA is 0.056 Gy/s/mA at the phantom surface and 0.023 Gy/s/mA at the center of the phantom with outer diameter (OD) of 4 cm. Figure 5c shows a depth dose rate plot using 160 kVp for three phantom sizes, 4 cm, 2 cm, and 1 cm. Compared to the largest 4-cm phantom, the smaller phantom size has higher dose rate at the center. Therefore, it is easier to achieve ultra-high dose rate in a smaller object.

### FLASH-RT anode current requirement

The required anode current for a given dose rate can be calculated based on Eq. (2). A dose rate of 100 Gy/s at the center of the 4 cm diameter cylindrical phantom requires about $4.5$ ampere total anode current. The x-ray generator will have a total power of 720 kW with 160 kVp. With 51 sources, the required anode current for each source ${I}_{FLASH}$ would be about 88 mA.

### Maximum dose of FLASH-RT delivered by a continuous (DC) beam

**Figure 6:**
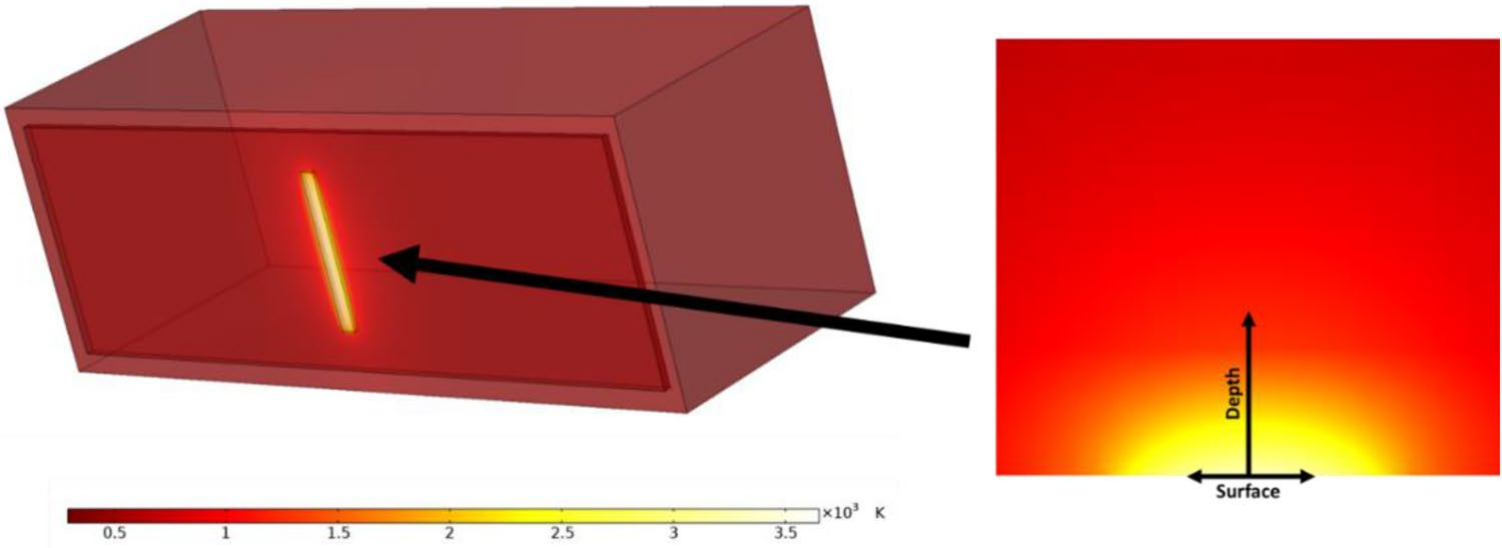
Temperature distribution of a 2 × 20 mm^2^ focal spot on the anode heated by a 160 keV 100 mA DC electron beam in FEA thermal analysis. The temperature distribution at the focal spot is shown in a cross-sectional view.

**Figure 7:**
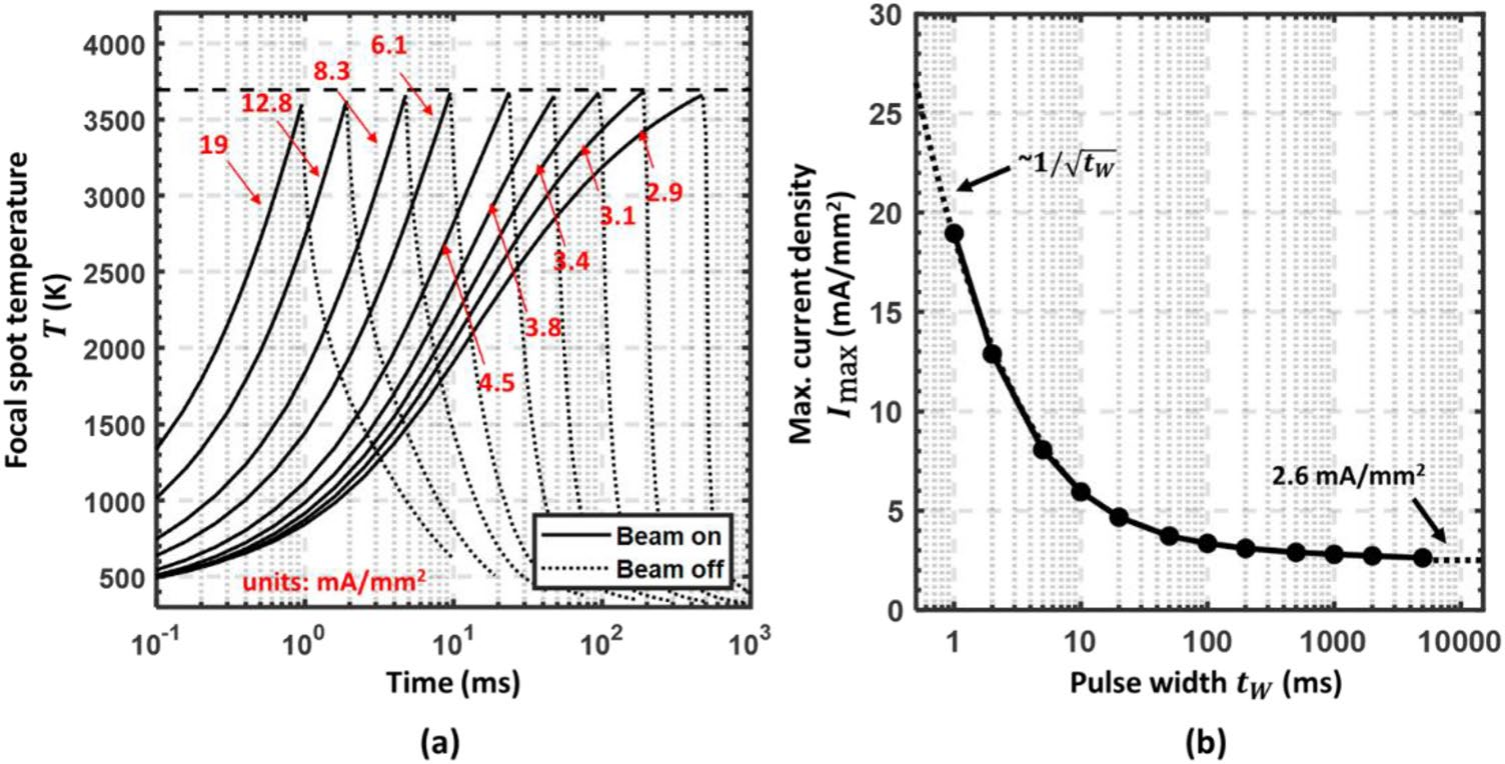
(**a**) Focal spot temperature vs. time for various pulse widths (${t}_{W}$= 1, 2, 5, 10, 25, 50, 100, 200 and 500 ms). The maximum incident current density ${I}_{\text{max}}/A$ used in each case is indicated with arrows. Solid lines indicate the temperature rise when the electron beam is on; dotted lines indicate the cooling process when the electron beam is off. The focal spot temperature $T$ are kept under the melting point of the tungsten target (3695 K). (**b**) Maximum incident current density (${I}_{\text{max}}/A)$ vs pulse width ${t}_{W}$. The curve is asymptotic to diffusive approximation ($\sim 1/\sqrt{{t}_{W}})$ for small ${t}_{W}$ and equilibrium ($\sim 2.6$ mA/mm^−2^) for large ${t}_{W}$.

Temperature change induced by a DC electron beam was obtained by transient thermal FEA performed in COMSOL (Fig. 6). The temperature $T$ at the center of the focal spot is the highest in the volume. Figure 7a shows the rise of $T$ over time for various temporal pulse width ${t}_{W}$ using the maximum incident current density ${I}_{\text{max}}/A$. For a given ${t}_{W}$, ${I}_{\text{max}}/A$ was chosen to be the maximum possible such that $T$ would remain below the melting point of tungsten (3695 Kelvin) for the full ${t}_{W}$. Figure 7b plots the maximum incident current density ${I}_{\text{max}}/A$ governed by the melting point of tungsten and ${t}_{W}$. As suggested by Oosterkamp^37^, ${I}_{\text{max}}/A$ at short ${t}_{W}$ is proportional to $1/\sqrt{{t}_{W}}$.

Based on the results in Fig. 7a, the maximum current density is 2.6 mA/mm^2^ for a DC beam at equilibrium, 3.1 mA/mm^2^ for a 200-ms beam, and 19 mA/mm^2^ for a 1-ms beam. If we consider the incident current requirement of each source to achieve 100 Gy/s dose rate with 51 sources that have been calculated in Section "FLASH-RT anode current requirement", the required minimum line focal spot area for each electron source is 33.8 mm^2^ for DC beam, 28.3 mm^2^ for a 200-ms beam, and 4.6 mm^2^ for a 1-ms beam. If the width of each line focal spot is 2 mm, the minimum length of each focal spot to achieve 100 Gy/s needs to be 17 mm, 15 mm, and 3 mm, respectively, which will be projected to effective focal spot sizes under 3 mm, 2.6 mm, and 0.5 mm respectively with 10° anode angle. In this study, we chose the focal spot area $A$ for each electron beam in the SAFI system to be 2 × 20 mm^2^, which will be sufficient to deliver a DC FLASH beam.

### Dose distribution and forward planning

**Figure 8:**
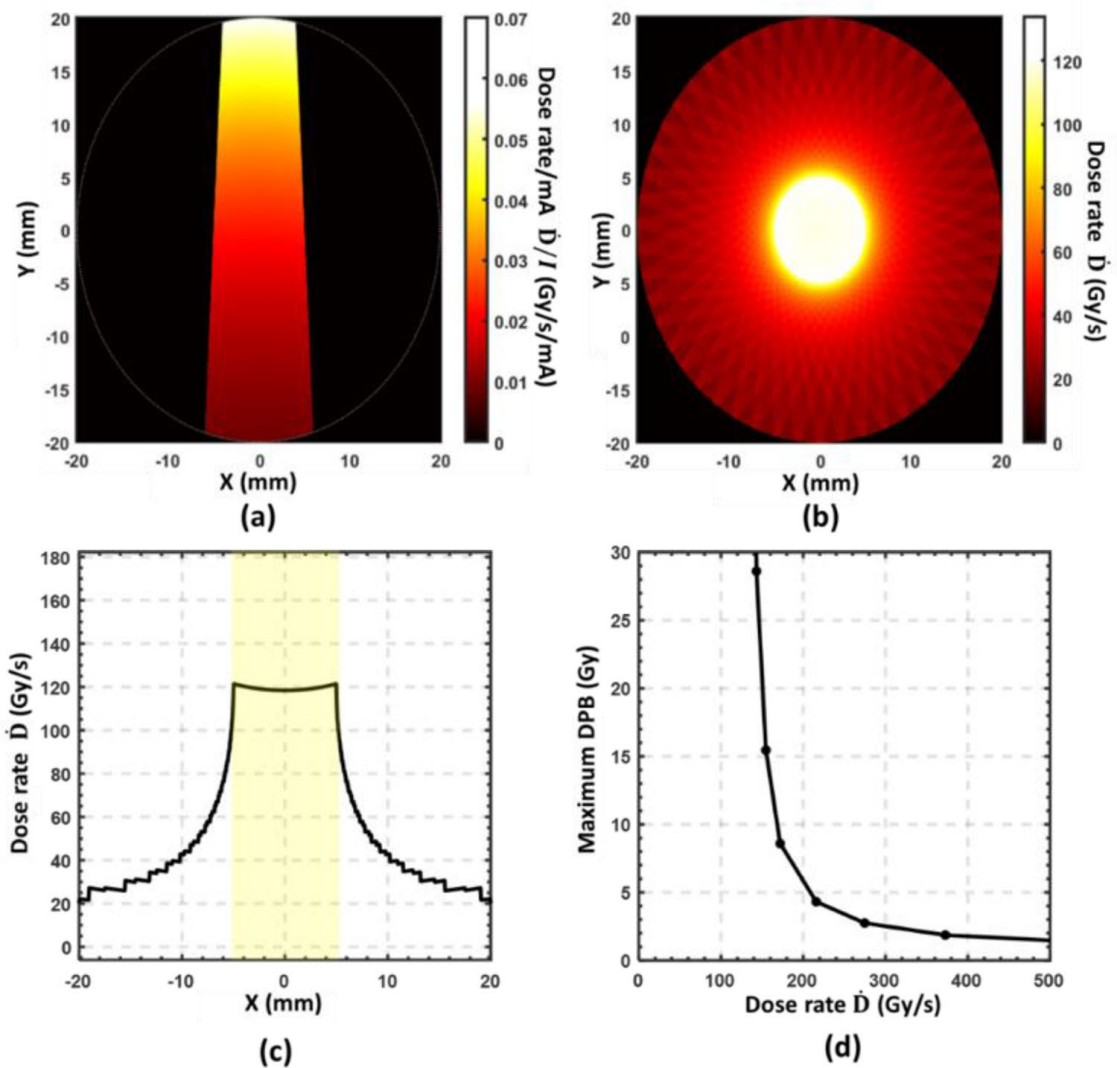
(**a**) Dose rate distribution in the cylindrical phantom of a single 160 kVp beam per unit anode current. (**b**) Total maximum dose rate distribution by 51 equally weighted DC beams each with a focal spot area 2 × 20 mm^2^. (**c**) Dose rate profile of the centerline in (**b**). The FLASH irradiation region is highlighted. (**d**) Maximum deliverable dose-per-beam (DPB) using focal spot area 2 × 20 mm^2^ at various dose rates.

In this study, we only considered a large 40-mm-diameter cylindrical water phantom. According to the depth-dose curves in Fig. 5, any smaller phantom will receive a higher dose rate. 3D dose distributions in the water phantom can be obtained by Monte Carlo calculation and converted to dose rate by Eq. (1). The focal spot size used in the simulation is 2 × 20 mm^2^. It is achievable in practice and demonstrated above to be suitable for achieving DC ultrahigh dose rates. Based on the ${I}_{\text{max}}/A$ results in Fig. 7, the maximal incident current ${I}_{\text{max}}$ for ${t}_{W}$ can be calculated. Figure 8a demonstrates the dose rate distribution by a single 160 kVp x-ray beam that was collimated to a 10 mm width at the center of the cylindrical phantom, as we presented in the design in Fig. 2. Figure 8b demonstrates the total dose rate distribution of all 51 beams. The DC anode current by each source is 104 mA (maximal for a 2 × 20 mm^2^ focal spot). The maximum dose rate in the target center will be about 120 Gy/s, and the largest dose rate deviation in the target region is within $\pm$ 2%. Figure 8c plots the dose rate profile through the phantom center. The maximum dose-per-beam (DPB) at the center of the phantom can be calculated for each dose rate by multiplying the pulse width ${t}_{W}$ with the corresponding maximum dose rate from Fig. 7b. Figure 8d plots the maximum DPB at various dose rates. For example, a dose of 10 Gy can be delivered using a dose rate up to 160 Gy/s in a 63 ms beam.

### Inverse FLASH-RT treatment planning

**Figure 9:**
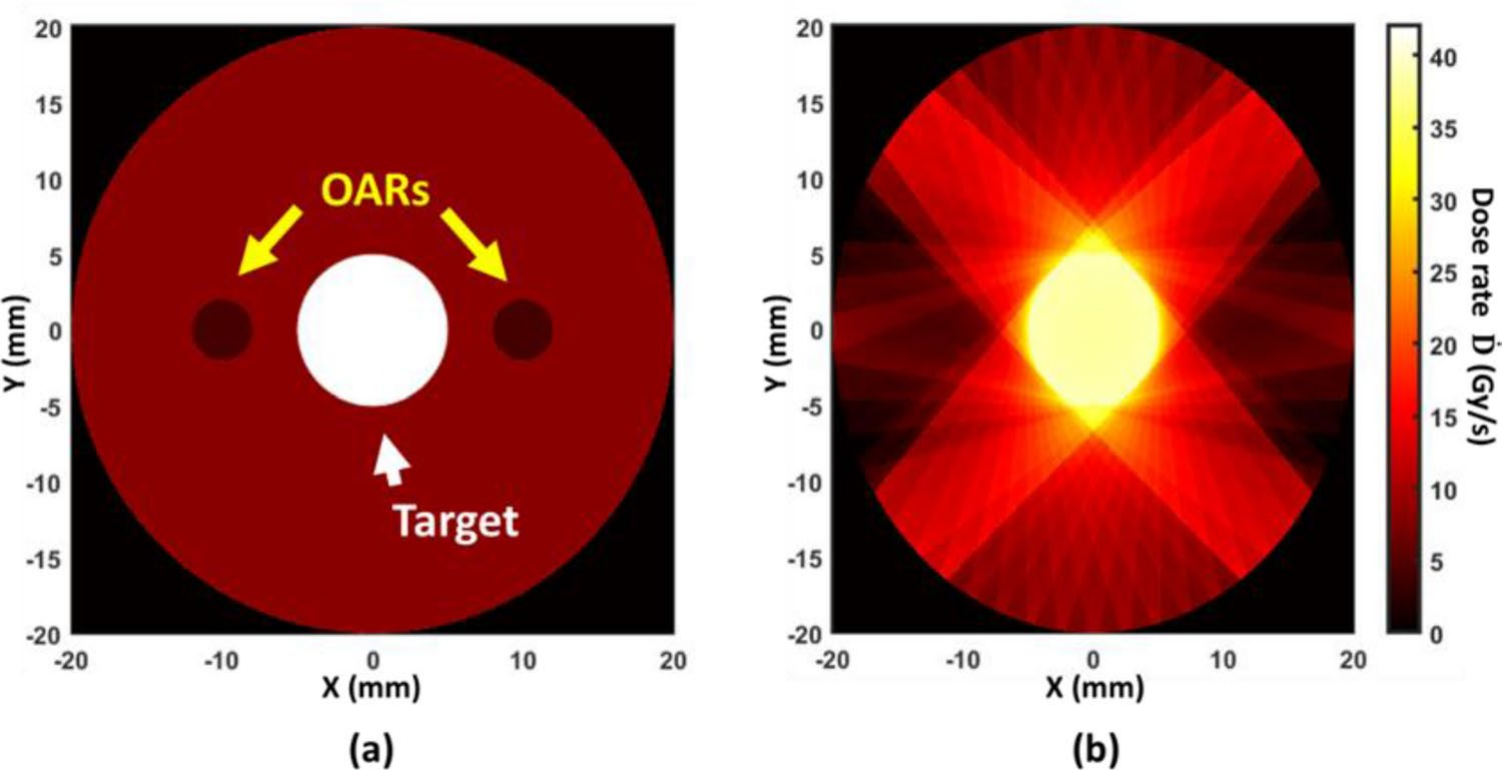
An example of inverse FLASH-RT planning. (**a**) The geometry with a 1 cm OD target in the middle of two 5 mm OD OARs. (**b**) Dose rate distribution obtained by inverse-optimization. The OARs doses are minimized while keeping the target DC dose rate at 40 Gy/s.

The weight of each beam can be modulated below the maximum beam power to achieve a more desirable dose distribution while still keeping over 40 Gy/s dose rate in the target. Figure 9a and b show the dose rate distribution of an inversely optimized treatment plan according to Eq. (5). A target of 10 mm in diameter is located in the middle of two OARs. The beam weights that contribute more dose to the OARs were minimized. The dose rate of the treatment target region can still maintain above 40 Gy/s in this example, whereas the average dose received in OAR regions is below 15% of the target dose.

### Electron optics design

**Figure 10:**
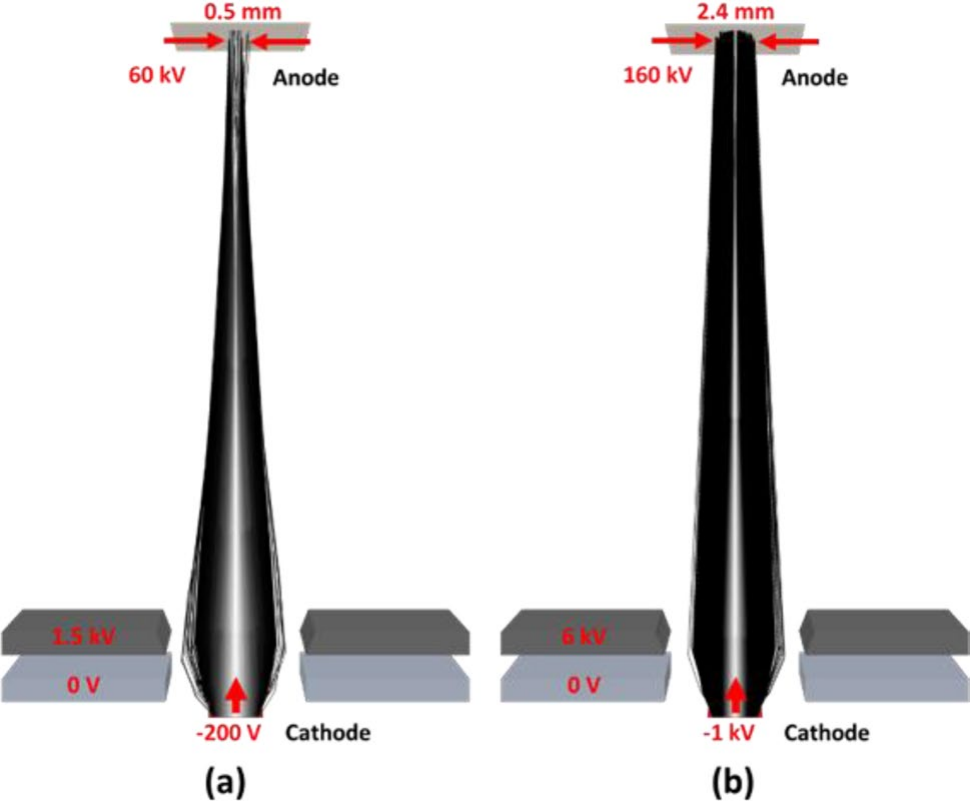
Example of electron beam optics of two operation modes: (**a**) inverse-geometry CT imaging mode and (**b**) FLASH-RT mode. Black lines represent the electron trajectories from a 2-mm wide electron source. The electron beam focal-spot width at the anode can be adjusted by the beam-focusing electrodes.

Figure 10 shows a simplified electron optics design that enables variable focal spot sizes for FLASH-RT and CT imaging modes. The electron beam focal-spot width can vary between 0.5 mm to 2.4 mm by changing the voltages of focusing electrodes. The 20 mm focal spot length is projected to 3.5 mm by the 10-degree anode angle. It can also be focused to a smaller focal spot size with a similar electron optics design. In FLASH-RT mode, the anode voltage is 160 kV, while a much lower anode voltage of 60 kV is used in the CT imaging mode.

### Inverse-geometry CT

**Figure 11:**
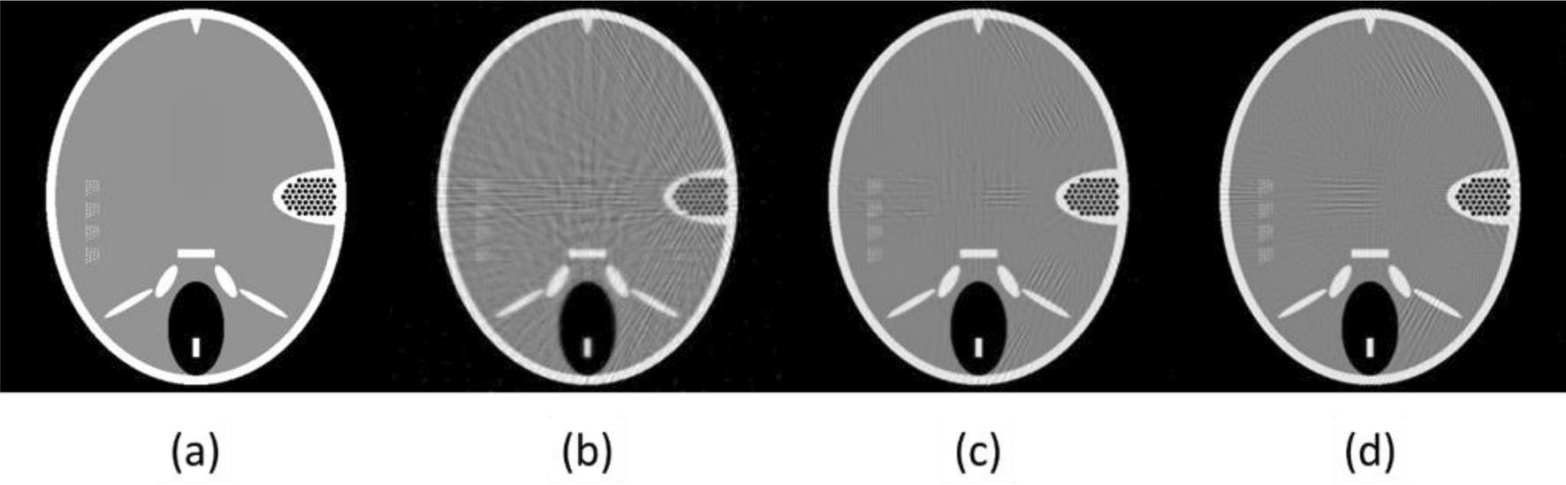
Simulation for inverse-geometry micro-CT. (**a**) The FORBILD head phantom. Reconstructed images using conjugate gradient algorithm with (**b**) 51 ($N=1$), (**c**) 102 (51 × 2, $N=2$), and (**d**) 153 (51 × 3, $N=3$) projection angles.

Projection data in inverse geometry CT was generated using the FORBILD numerical head phantom (shown in Fig. 11a) and reconstructed using an iterative reconstruction algorithm. The reconstructed images of stationary and rotating SAFI systems with different projection angles are shown in Fig. 11b–d. Due to insufficient views, stationary-source imaging ($N$ = 1) has lower resolution and artifacts due to high smoothness constraints, although it may still be good enough for image guidance. The rotating system is simulated using $N$ = 2 and $N$ = 3 rotation steps that produce 102 and 153 projection angles, respectively. The SAFI system can produce images with sufficient resolution for treatment planning with additional rotation steps.

## Discussion

Pre-clinical studies of new radiation treatment techniques with small animal irradiators are of paramount importance for the advancement of human radiotherapy. It is important that dedicated platforms can administer radiation doses to animals in a manner comparable to human treatments in radiation oncology clinics. While proton accelerators and linacs can potentially deliver conformal FLASH-RT to human patients, current small animal FLASH-RT modalities based on proton accelerators or dedicated linacs have various limitations, such as accessibility to the machines and limited penetration depth of the electron beams. Most current small animal x-ray FLASH-RT solutions are not suitable for conformal doses to deep-seated targets. Our design of SAFI based on distributed x-ray source technology potentially can deliver a highly conformal dose with UHDR and allow broad pre-clinical studies of FLASH-RT.

The choice of 51 sources of SAFI is based on the tradeoff between maximum field size and dose rate. With 51 sources turned on simultaneously, the current design allows treating a target size of 1 cm in diameter. Further increasing the number of sources is possible, but the field size would then be reduced to accommodate the MAC. Further shortening the distance between the MAC and the anode would allow treating a larger target. Because the MAC is installed in the air, the anode on high voltage has to be a certain distance from the MAC to provide sufficient dielectric insulation. Grounding the anode will allow further reduction of distance from the focal spot to the MAC, but the source scanning control would be difficult to design with the beam scanning electronics on negative high voltage. The maintenance of 51 sources in SAFI would be similar to the previously developed 48-source x-ray tube for imaging^30,31^, including cathode emission and high-voltage conditioning tests.

Rotating anode is broadly used in high-power x-ray sources. In principle, SAFI may employ a rotating anode if necessary. Based on our simulation, a fixed anode with 51 focal spots could produce sufficient x-ray flux for FLASH-RT. A fixed anode configuration reduces both the complexity and the cost of the system. The anode can be made of oxygen-free copper brazed with a tungsten target. Because a single FLASH-RT operates for a very short time, the temperature rise of the anode will be only a couple hundred degrees after a FLASH-RT delivery. Thus, active cooling is not necessary for the SAFI system.

During FLASH-RT delivery, the total anode currents for 51 cathodes will be a few amperes, and the instantaneous power will be as high as a few hundred kilowatts. A high-voltage power supply with high power is not easily accessible. A pulse-discharge capacitor can be used as the power supply of the SAFI. The capacitor may be charged to 160 kV slowly before treatment and discharged during the treatment. The voltage drop can be compensated in treatment planning. In the case of conventional dose-rate RT, the total anode currents will be reduced to a few milli-amperes. Due to the large focal spot area, the maximum target temperature will be far from its limits and can continuously operate for over an hour, as shown in Fig. 7.

We demonstrated that 160 kVp is sufficient for small animals. To treat larger animals such as dogs and cats, a higher kVp is desired. Not only can higher kVp x-ray penetrate large animal bodies, but the efficiency of Bremsstrahlung x-ray production improves with electron beam energy. However, the SAFI system, as well as other distributed x-ray sources, employs a unipolar design with cathodes close to ground potential due to the complicated source switching control electronics. It will be challenging to achieve greater than 160 kV in unipolar design.

The majority of clinical RT machines, including commercial linacs and proton therapy systems, deliver pulsed radiation beams. Many FLASH radiobiological questions require investigating various microstructure parameters of the beam used in dose delivery^15^. For example, many studies claimed the benefits of delivering doses in pulses with a mean dose rate higher than FLASH^11,38^. Studies also showed that the total dose and beam-pulse configuration, in addition to the mean dose rate, have various biological impact^1,4,7,17,18^. Our SAFI system can deliver FLASH doses either in one DC pulse or in multiple shorter pulses. If using a 2 × 20 mm^2^ focal spot, a shorter pulse width allows a much higher instantaneous dose rate. In this case, however, the anode current and pulse width need to be carefully controlled to avoid damaging the tungsten target.

Finally, this study emphasizes the instrumentation of the design, beam requirements, and feasibility. The treatment planning study is rather simplistic but enough to show the capability of delivering conformal FLASH irradiation in an animal smaller than a 4-cm cylindrical water phantom. In this study, we demonstrated beam-weight optimization for inverse treatment planning, while beam modulation using compensator-based or aperture-based methods is also suitable in the SAFI with modification of the collimator design. Moreover, the dose threshold effect in non-target volume and animal setup can also be addressed in treatment planning. Including these considerations, future studies will develop a more complex treatment planning methodology.

## Conclusions

Our small animal FLASH irradiator (SAFI) with 51 sources based on distributed x-ray source can deliver UHDRs (up to 120 Gy/s) in treatment volume for pre-clinical FLASH-RT studies, as well as conventional dose rates for comparative studies. It is also capable of performing inverse-geometry micro-CT imaging with a rotatable add-on detector.

## Data Availability

Data is available from the corresponding author upon reasonable request.
